# Non-alcoholic fatty liver disease (NAFLD): a tale of fat and sugar?

**DOI:** 10.1186/1755-1536-6-14

**Published:** 2013-07-18

**Authors:** Lisa Longato

**Affiliations:** 1 UCL Institute for Liver and Digestive Health, Royal Free Hospital, U3rd Floor, Rowland Hill Street, London NW3 2PF, UK

**Keywords:** Fructose, High-fat diet, High-fructose corn syrup, Mice, NAFLD, Non-alcoholic steatohepatitis, Rats, Western diet

## Abstract

The global diffusion of the so-called Western diet, which is enriched in fat and carbohydrates, such as fructose, has been proposed to be an underlying cause of the increased prevalence of metabolic conditions, including non-alcoholic fatty liver disease (NAFLD). This Smart Card summarizes the main metabolic and hepatic histological features of rodent models fed with diets combining high fat and fructose.

## Introduction

Non-alcoholic fatty liver disease (NAFLD), a condition regarded as the hepatic manifestation of the metabolic syndrome, currently represents the most common cause of chronic liver disease [1]. The condition ranges from simple hepatic fat accumulation (steatosis) to non-alcoholic steatohepatitis, where fat is accompanied by hepatocyte injury, and necroinflammation. This condition poses an increased risk of cirrhosis and hepatocellular carcinoma [1]. The dramatic increase in prevalence of obesity, metabolic syndrome, and NAFLD has been linked to the global diffusion of the Western diet, characterized by excess caloric intake due to increased consumption of processed food and beverages, coupled with a more sedentary lifestyle [2,3]. This has led to a significant increase in sucrose and high-fructose corn syrup consumption, both of which contain similar amounts of glucose and fructose [4]. In the USA, for example, fructose consumption has more than doubled in the last three decades [3]. Excessive fructose consumption has been linked to an increased prevalence of metabolic diseases and growing evidence suggests that it may also contribute to the development and severity of NAFLD by exacerbating fat deposition, inflammation, and, possibly fibrosis [5]. Mechanistically, fructose may contribute to NAFLD by promoting *de-novo* lipogenesis, insulin resistance, oxidative stress, bacterial overgrowth, and inflammation [3-7]. The mechanisms responsible for transition to non-alcoholic steatohepatitis are still not completely understood, in part because of the scarcity of animal models that can fully replicate both the histological and metabolic features of human non-alcoholic steatohepatitis [8]. As fructose is likely to act as a dietary ‘second hit’ [5], effort has recently been put into developing novel experimental models to recapitulate the Western diet by combining high-fat or high-energy diets and fructose. The aim of this Smart Card is to provide a synthetic and exhaustive source for rapid consultation of the currently proposed rodent models of diets combining high fat and fructose, summarizing the metabolic and hepatic consequences of such combinations (Table 1).

**Table 1:** Metabolic and hepatic features of rodent models fed with diets combining high fat and fructose

**Name of model, species, or strain**	**Diet**	**Weeks**	**Metabolic features**^**a**^	**NAFLD features**^**a**^	
ALIOS diet (long-chain saturated trans fat)	Standard chow (13.6% kcal from fat (soybean oil (15% saturated fatty acids, 23% monounsaturated fatty acids, 61% polyunsaturated fatty acids)) + gel-water;	16	↑ body weight, ↑ liver weight;	↑ alanine aminotransferase, ↑ aspartate aminotransferase;	[9]
C57BL/6 J mouse	**Alios diet: 45% kcal from fat (30% from partially hydrogenated vegetable oil (28% saturated fatty acids, 57% monounsaturated fatty acids, 13% polyunsaturated fatty acids) + high-fructose corn syrup equivalent (55% fructose, 45% glucose by weight) (42 g/l as gel-water)**		↑ liver triglycerides;	macrosteatosis (zones 1 and 2), microsteatosis (zone 3);	
			↑ insulin, ↑ leptin;		
			= adiponectin;	lobular inflammation;	
			= triglycerides, ↑ cholesterol (plasma)	possible ballooning or Mallory-Denk body fibrosis: not observed	
Modified ALIOS	Chow diet;	16	↑ body weight, ↑ liver weight;	↑ alanine aminotransferase;	[10]
(medium chain saturated fatty acids)	High-fat diet (58% kcal from fat);		↑ glucose/insulin (fasting);	steatosis (micro, macro);	
C57BL/6 J mouse	**High-fat diet + water with high-fructose corn syrup equivalent (55% fructose and 45% sucrose by weight (42 g/l))**		↑ HOMA-IR;	lobular inflammation, ↑ apoptosis;	
			↑ liver triglycerides	ballooning: not assessed or not reported;	
				↑ fibrosis	
Fast-food mouse	Standard chow (13% kcal from fat (1% saturated fatty acids) + high-fructose corn syrup in water (42 g/l));	25	↑ body weight, ↑ liver weight;	↑ aspartate	[8]
C57BL/6 J mouse	High-fat diet (60% kcal from fat (1% saturated fatty acids) + high-fructose corn syrup in water (42 g/l));		↑ glucose, insulin (fasting);	aminotransferase;	
	**Fast-food diet (40% kcal from fat (12% saturated fatty acids) +2% cholesterol + high-fructose corn syrup in water (42 g/l))**		↑ HOMA-IR;	steatosis: panacinar;	
			↓ adiponectin;	(++ macro, + micro);	
			↑ cholesterol (plasma)	intra-acinar inflammation;	
				ballooning;	
				fibrosis: peri-sinusoidal, -cellular	
High-fat diet + fructose (water)	Control diet (4.8% fat);	8	↑ body weight, = liver weight;	Liver function tests: not assessed or not reported;	[11]
C57BL/6 J mouse	**60% fat diet + 30% fructose in water**		↑ glucose, ↑insulin (fasting);	centrilobular fat vacuolation;	
			↑ HOMA-IR, ↑ GTT-AUC;	(↑ Oil Red O);	
			↑ leptin;	fibrosis: not assessed or not reported	
			↑ triglycerides (plasma), ↑ cholesterol (plasma)		
High-fat diet + sucrose	Control group (control);	12	↑ body weight;	Liver function tests: not assessed or not reported;	[12]
Wistar rats	**High fat, high sucrose**		=glucose, = insulin;	↑ macrosteatosis	
			↑ cholesterol, = triglycerides (plasma);		
			↑ leptin		
High-fat diet + fructose (water)	Control diet;	8	↑ body weight, ↑ liver weight;	Liver function tests: not assessed or not reported;	[13]
C57BL6/J mouse	**High-fat diet (72% fat (corn oil/lard); 28% protein, <1% carbohydrates) + 21% fructose in water**		= liver triglycerides, ↑ GTT-AUC;	↑ macrosteatosis;	
			=glucose, ↑ insulin (fasting)	fibrosis: not assessed or not reported	
High-fat diet + fructose (water)	Standard chow (5% kcal from fat, 18% proteins, 77% carbohydrates)	12	↑ body weight;	↑ alanine aminotransferase;	[14]
Sprague–Dawley rat	Standard chow + fructose in water (30% w/v);		glucose =, ↑ insulin;	ballooning + mild steatosis;	
	High-fat diet (58% kcal from fat, 18% protein, 24% carbohydrates)		↑ triglycerides, ↑ cholesterol (plasma)	no inflammation;	
	**High-fat diet + fructose in water (30% w/v)**			rare fibrosis	
High-fat diet + fructose (water)	Standard chow (24% protein, 11% fat, 65% carbohydrates (% by weight));	15	↑ body weight, ↑ liver weight;	↑ alanine aminotransferase, ↑ aspartate aminotransferase;	[15]
Wistar rats	**High-fat diet [26% fat, 17% protein, 4% cholesterol, 53% carbohydrates) + 10% (w/v) fructose in water**		↑ liver triglycerides, ↑ HOMA-IR;	↑ Oil Red O;	
			↑ insulin, = glucose (fasting);	fibrosis: not assessed or not reported	
			↑ leptin, ↓ adiponectin		
High-fat diet + fructose (water)	Normal chow (4% fat);	48	↑ body weight, ↑ liver weight;	Liver function tests, fibrosis: not assessed or not reported;	[16]
Sprague–Dawley rats	**High-fat diet (60% kcal from fat) + 10% fructose in water**		= glucose (fasting), ↑ GTT-AUC;	macroscopic signs of steatosis	
			↑ triglycerides, = cholesterol (plasma)		
High-fat diet + fructose (water)	Low-fat diet: 10% kcal from fat;	10	↑ Body weight, ↑ liver weight;	↑ alkaline phosphatase, = alanine aminotransferase;	[17]
Sprague–Dawley rats	**Western diet: high-fat diet (45% kcal from fat (soybean oil, lard)) + high-fructose corn syrup-55 (55% fructose, 45% glucose diluted with water to 12.5%)**		↑ GTT-AUC/↑ leptin;	↑ liver fat score, ↑ NAFLD activity score;	
			= triglycerides (plasma);	= lobular inflammation, = ballooning;	
			↓ cholesterol (plasma)	fibrosis: no	
High-fat diet + fructose (water)	Cornstarch diet;	16	↑ body weight, = liver weight;	↑ alanine aminotransferase /↑ aspartate aminotransferase;	[18]
Wistar rats	**High-fat, fructose diet (52% carbohydrate, 24% fat, 25% fructose in drinking water)**		↑ basal glucose;	↑ fat vacuoles;	
			↑ plasma triglycerides (plasma)	↑ portal inflammation;	
				fibrosis: portal	
High-fat diet + fructose (chow)	Standard chow;	16	↑ body weight, ↑ liver weight;	↑ alanine aminotransferase/ aspartate aminotransferase;	[19]
C57BL/6 J mice	**High-fat, high-fructose diet (in solid diet)**		↑ HOMA-IR;	micro/macro-steatosis (pericentral);	
			↑ insulin, glucose (fasting);	fibrosis: not assessed or not reported	
			↑ liver triglycerides		
High-fat diet + fructose (chow)	Control group (cornstarch diet);	5	= body weight;	= alanine aminotransferase;	[20]
	High fructose (70% by weight);		= plasma triglycerides;	some macrosteatosis;	
Wistar rats	High sucrose (70%);		= glucose;	inflammation: =lobular, = portal;	
	High fat (15%);		= liver triglycerides	fibrosis: no change	
	**High fat (15%), high fructose (50%)**				
High-fat diet + fructose (chow)	Low-fat, high-carbohydrate (cornstarch) diet;	32	↑ body weight, ↑ liver weight;	↑ alanine aminotransferase, alkaline phosphatase;	[21]
Wistar rats	**High-carbohydrate (fructose/sucrose), high-fat diet**		↑ visceral adiposity;	= liver triglycerides, lipid deposition;	
			↑ % body fat	inflammation;	
				fibrosis: not assessed or not reported	
High-fat diet + fructose (chow + water)	Control: cornstarch diet;	16	↑ body weight, ↑ liver weight;	↑ alanine aminotransferase, ↑ aspartate aminotransferase, ↑ alkaline phosphatase;	[22]
Wistar rats	**High-carbohydrate, high-fat diet (including condensed milk (39.5%), beef tallow (20%), and fructose (17.5%)) + 25% fructose in water**		↑ glucose, = insulin (fasting);	↑ macrosteatosis;	
			↑ GTT-AUC;	↑ inflammation;	
			↑ cholesterol, ↑ triglycerides (plasma)	mild portal fibrosis	

^a^All indicated changes refer to the experimental group in bold compared with either a control diet or other listed groups.

## Abbreviations

GTT-AUC: Glucose tolerance test: area under the curve; HOMA-IR: Homeostasis model of assessment - insulin resistance; NAFLD: Non-alcoholic fatty liver disease; w/v: Weight by volume.

## Competing interests

The author declares that she has no competing interest.
